# Arginine Consumption by the Intestinal Parasite *Giardia intestinalis* Reduces Proliferation of Intestinal Epithelial Cells

**DOI:** 10.1371/journal.pone.0045325

**Published:** 2012-09-19

**Authors:** Britta Stadelmann, María C. Merino, Lo Persson, Staffan G. Svärd

**Affiliations:** 1 Department of Cell and Molecular Biology, Biomedical Centre Uppsala, University of Uppsala, Uppsala, Sweden; 2 Instituto de Investigación Médica Mercedes y Martin Ferreyra-Conicet, Córdoba, Argentina; 3 Department of Experimental Medical Science, Biomedical Centre Lund, University of Lund, Lund, Sweden; Technion-Israel Institute of Technology Haifa 32000 Israel, Israel

## Abstract

In the field of infectious diseases the multifaceted amino acid arginine has reached special attention as substrate for the host´s production of the antimicrobial agent nitric oxide (NO). A variety of infectious organisms interfere with this part of the host immune response by reducing the availability of arginine. This prompted us to further investigate additional roles of arginine during pathogen infections. As a model we used the intestinal parasite *Giardia intestinalis* that actively consumes arginine as main energy source and secretes an arginine-consuming enzyme, arginine deiminase (ADI). Reduced intestinal epithelial cell (IEC) proliferation is a common theme during bacterial and viral intestinal infections, but it has never been connected to arginine-consumption. Our specific question was thereby, whether the arginine-consumption by *Giardia* leads to reduced IEC proliferation, in addition to NO reduction. *In vitro* cultivation of human IEC lines in arginine-free or arginine/citrulline-complemented medium, as well as in interaction with different *G. intestinalis* isolates, were used to study effects on host cell replication by MTT assay. IEC proliferation was further analyzed by DNA content analysis, polyamine measurements and expressional analysis of cell cycle regulatory genes. IEC proliferation was reduced upon arginine-withdrawal and also in an arginine-dependent manner upon interaction with *G. intestinalis* or addition of *Giardia* ADI. We show that arginine-withdrawal by intestinal pathogens leads to a halt in the cell cycle in IECs through reduced polyamine levels and upregulated cell cycle inhibitory genes. This is of importance with regards to intestinal tissue homeostasis that is affected through reduced cell proliferation. Thus, the slower epithelial cell turnover helps the pathogen to maintain a more stable niche for colonization. This study also shows why supplementation therapy of diarrhea patients with arginine/citrulline is helpful and that citrulline especially should gain further attention in future treatment strategies.

## Introduction

Arginine is a conditionally essential amino acid, implying that it is essential under non-physiological conditions or disease, as well as in growing individuals. In healthy adults, arginine synthesized through catabolic reactions in intestine and kidney is fully sufficient [1]. Arginine is a multifaceted amino acid, not only being important as a component of proteins, but also as a precursor for a variety of other molecules [1]. Accordingly, arginine has received increasing attention over the last decades, playing various roles in different disease states (such as cardiovascular disease, diabetes, etc.). In the field of infectious diseases, major focus has been put on the role of arginine as a substrate for the host´s production of the antimicrobial agent nitric oxide (NO). A variety of infectious organisms, such as *Mycobacterium, Trypanosoma, Helicobacter, Schistosoma and Salmonella spp*., were shown to interfere with this part of the host immune response by reducing the availability of arginine, usually by up-regulation of host arginases [2], [3]. This prompted us to further investigate additional roles of arginine during pathogen infections, focusing on intestinal infections. As a model we used the protozoan parasite *Giardia intestinalis* that actively consumes the amino acid arginine.

*G. intestinalis* (referred to as *Giardia*) is an important cause of diarrheal disease and is responsible for approximately 250 million cases of human giardiasis per year [4], [5]. *Giardia* consists of two life cycle stages: the infectious stage, represented by cysts, is able to survive in water and moist environment. Upon ingestion by a host, cysts are activated during passage of the acidic milieu in the stomach and undergo excystation in the duodenum. This results in release of proliferating trophozoites, establishing an intestinal infection [6]. Symptoms of the multifactorial disease giardiasis include watery diarrhea, nausea, vomiting and epigastric pain [5]._ENREF_9 About half of the infections are asymptomatic, but may also develop into a chronic state [5]. There is a big genetic variability in *Giardia* and 8 different genotypes/assemblages (A-H) have been identified [5]. Human infections are caused by assemblage A and B, but these two display only 78% nucleotide identity [7], [8]. As energy sources *Giardia* can use glucose, but preferably degrades arginine via the arginine dihydrolase pathway that is classically described in prokaryotes [9]. Within this pathway, arginine is converted into citrulline and ammonia by arginine deiminase (ADI), further into ornithine and carbamoyl phosphate by ornithine carbamoyltransferase (OCT) and finally into ammonia and CO_2_ by carbamate kinase (CK), leading to the direct generation of ATP via substrate level phosphorylation. According to calculations made by Schofield et al, *Giardia* can produce 7–8 times more energy from arginine than from glucose and *Giardia* uses arginine as the major energy source [9]. Upon *in vitro* interaction with IECs, *Giardia* releases several proteins within the first 30 minutes of interactions, among them ADI and OCT [10]. This puts an additional stress on the putative importance of arginine in the host-pathogen interaction of giardiasis, since these two released enzymes can lead to further local arginine depletion. A possible role of arginine-depletion in giardiasis was hypothesized by Eckmann et al [11] who showed that *in vitro* infection of IECs leads to a reduced NO production, as already mentioned for other pathogens [2]. Later it was shown that addition of ADI expressed in *E. coli* could reduce NO production by IECs [10]. Other putative consequences of local arginine depletion on host IECs have not yet been taken into account, also not in terms of pathogenicity.

IECs are protected against pathogenic infection through various mechanisms such as epithelial integrity, epithelial cell turnover, intestinal immune responses, commensal microflora and mucus layer [12]. Since previous studies made by Roxström-Lindquist et al [13] highlighted a reduction of genes of cell proliferation upon *Giardia*-interaction in IECs and Laenerts et al [14] detected a reduced expression of cell proliferation proteins upon arginine withdrawal in pre-confluent Caco2 IECs, we were interested in assessing the role of arginine and epithelial cell turnover during intestinal infection. We applied an *in vitro* model based on human IECs and their growth in arginine-free and arginine-complemented medium, as well as their growth upon interaction with the arginine-consuming parasite *Giardia* and assessed the *in vitro* effects of arginine depletion with regards to cell cycle progression. Hereby this study is also relevant for intestinal infections by other pathogens that are known to interfere with host arginine usage.

We could show that arginine consumption by pathogens, such as *G. intestinalis*, during host-cell interactions results in growth arrest of IECs. This, in combination with a reduction in NO production, shows that arginine plays a crucial role during intestinal infections. Thus, our specific aim, to see whether arginine-consumption of *Giardia* can lead to reduced IEC proliferation, could be achieved. In addition, we showed that reduced IEC proliferation could be counteracted by treatment with the arginine-metabolite citrulline that will be applied in future *in vivo* studies.

## Materials and Methods

### Reagents and Cell Culture

If not stated otherwise, all chemicals and reagents were purchased from Sigma Chemical Co, USA.

Human intestinal epithelial cell (IEC) lines Caco2 clone TC7 and HCT-8 (ATCC CCL-244) were maintained as described before [10] in complete DMEM. TC7 cells were cultivated for 21 d with medium changes 2 × a week to allow differentiation of these cells [10].

*Giardia* trophozoites (strain WB, clone C6 (ATCC30957); strain GS, clone H7 (ATCC50581); strain P15 [7]) were maintained in 10 mL TYI-S-33 medium supplemented with bile according to Keister et al [15]. For interaction, trophozoites were washed in cold PBS 2x, diluted in complete DMEM (high-glucose DMEM with 10% fetal bovine serum (Gibco®, Invitrogen, Paisley, UK), 4 mM L-glutamine, 1 x MEM non-essential amino acids, 160 µg/mL streptomycin and 160 U/mL penicillin G) and added to IECs at indicated numbers/ratios. All incubations were performed at 37°C, 5% CO_2_, humid atmosphere.

### Production and Purification of Recombinant Arginine Deiminase (ADI) and Ornithine Carbamoyltransferase (OCT) in *Giardia*

ADI (http://giardiadb.org/giardiadb/: GL50803_112103) and OCT (http://giardiadb.org/giardiadb/: GL50803_10311) were expressed in and purified from *Giardia* and activities determined as described in Jerlström-Hultqvist et al [16].

### Amino Acid Analysis

To assess amino acid changes in the medium of *Giardia* trophozoites interacting with IECs, TC7 cells were seeded to 30′000/well in 96 well-plates (Corning, New York, USA) in complete DMEM. After letting the cells attach overnight, *Giardia* trophozoites were added at a ratio of 1∶6 (human cell:parasite). Medium samples were taken after 1, 2 and 24 h of interaction and compared to corresponding controls. Amino acid analysis was performed by the Amino Analysis Centre, University of Uppsala, Biomedical Centre, Uppsala, Sweden.

### IEC growth measurements

#### Under low arginine-conditions

For assessment of IEC numbers under different growth conditions, cells were used in proliferative and differentiated states. To evaluate effects in proliferative state, cells were seeded in triplicates into 96 well-plates in arginine-free medium (RPMI 1640 (with L-glutamine, without arginine, leucine, lysine or phenol red) supplemented with 10% fetal bovine serum, 160 µg/mL streptomycin, 160 U/mL penicillin G, 0.4 mM L-Lysine and 0.38 mM L-Leucine) or in arginine-free medium supplemented with 0.4 mM L-Arginine or 0.4 mM L-Citrulline. After 1–5 d (24 h interval) cell numbers were measured by MTT assay (described below) or RNA extracted by the RNeasy® Mini Kit (QIAGEN AB, Sollentuna, Sweden) according to manufactureŕs instructions. cDNA generation and expression analysis was performed as described below. Differentiated TC7 cells were grown and measured accordingly.

#### *Giardia* – IEC interaction

To determine the impact of *Giardia* trophozoites on IEC number, interactions with IECs were set up as described for the amino acid analysis. To revert arginine-dependent effects of *Giardia*, arginine was added to 0.4 mM final concentration after 2 and 18 h. After 48, 72 and 96 h of interaction, relative cell numbers were assessed by MTT assay.

#### ADI-transfected *Giardia* – IEC interaction

To determine the impact of ADI-transfectants, IECs were seeded at 30′000/well in complete DMEM in 96 well-plates. After overnight attachment, *Giardia* trophozoites (WB and ADI-transfectants, generated as described above), were added at a ratio of 1∶5 (human cell:parasite) in triplicates. After 4 d of incubation, relative cell numbers were assessed by MTT assay.

### IEC and Addition of ADI and OCT

The effects of ADI and OCT were assessed on proliferating and differentiated IECs. 10′000 HCT-8 cells/well or 30′000 TC7 cells/well were seeded in 96 well-plates in complete DMEM. After incubation overnight, ADI was added to 27.5 U/L, OCT to 43.5 U/L, as well as corresponding buffer and boiled protein or medium controls and restimulated after 16 h. Additionally, arginine was added to 0.4 mM after 2 and 18 h. All stimulations were set up in triplicates. Relative cell numbers were determined at different time points (as indicated) by the MTT assay. Differentiated TC7 cells were stimulated and measured accordingly.

### MTT Assay

To quantify the amount of metabolically active cells in various setups, 10% of MTT solution (5 mg/mL) was added to cells in growth medium, following incubation at 37°C for 5 h. Subsequently, cells were lysed by replacement of medium by the same amount of DMSO and dissolved for 30 minutes at 37°C. The OD was measured at 540 nm in a Multiskan MS Plate Reader (Labsystems, Finland).

### Gene Expression Analysis

#### IEC gene expression under low arginine-conditions

For assessment of gene expression of IECs under low arginine-conditions, IECs were cultured either close to confluence (proliferating) or 21 d post confluence in complete DMEM. After being washed 1 × in PBS, medium was changed to arginine-free RPMI or arginine-free medium supplemented with 0.4 mM L-arginine. Samples for RNA extraction were taken after 0, 1.5, 3, 6, 24, 30 and 48 h. Therefore, supernatant medium was removed and human IECs were washed 1 × in PBS before being taken up in 1 mL TRIZOL® (Invitrogen) and stored at −20°C until further RNA extraction.

#### *Giardia* – IEC interaction: gene expression

For assessment of gene expression of *Giardia* infected human IECs, these were cultured close to confluence (proliferating). Before addition of parasites, IECs were washed 1 × in DMEM. Subsequently, 7.1 × 10^6^ parasites were added to each bottle. Samples for RNA extraction were taken after 0, 1.5, 3, 6 and 24 h. Therefore, culture flasks were incubated on ice for 10 minutes to let parasites detach, supernatant medium was removed and IECs were washed 2 × in cold PBS before being taken up in 1 mL TRIZOL® (Invitrogen) and stored at −20°C until further RNA extraction.

#### RNA sample preparation and qPCR

To assess the expression status of different genes in IECs on RNA level, RNA was extracted from respective interaction samples according to standard TRIZOL protocol. Contaminating gDNA was removed by use of TURBO DNA-free™ (Ambion, Austin, TX) according to manufactureŕs instructions. cDNA was generated by use of the High-Capacity cDNA Reverse Transcription Kit (Applied Biosystems, Foster City, CA) applying 2 µg RNA/reaction. qPCR was performed using Power SYBR® Green PCR Master Mix (Applied Biosystems) according to manufactureŕs instructions. Primers were designed by use of the softwares Primer Express 3.0 (Applied Biosystems) and NetPrimer (PREMIER Biosoft, Palo Alto, CA) and are given in Table S1. PCR reactions were run in quadruplicates on a 7300 Real Time PCR System machine (Applied Biosystems) with the standard program. Human GAPDH was used as a reference gene.

### DNA Content Analysis

DNA content measurements in IECs were performed by propidium iodide staining and subsequent analysis by flow cytometry. In short, IECs were set up for interaction with different *Giardia* isolates as described above. At timepoints indicated, *Giardia* trophozoites were removed by chilling culture flasks on ice for 10 minutes, subsequent 2 × washing of IECs with ice-cold PBS and detaching of IECs by trypsinization at 37°C for 10 minutes. Cells were centrifuged at 500 x g, 4°C, 10 minutes and taken up in 200 µl cold PBS. Under vortexing, the cells were added to 4 mL of ice-cold 70% ethanol and fixed for a minimum of 4 h at 4°C. Subsequently, cell clumps were removed by filtration through a 40 µM cell strainer (BD Falcon™, Franklin Lakes, NJ, USA). The cells were centrifuged as before, taken up in 207 µl PBS and stained by addition of propidium iodide (Millipore, Billerica, MA, USA) to 40 µg/mL and RNase treated by addition of RNase A (Fermentas AB, Helsingborg, Sweden) to 100 µg/mL. Cells were kept at 4°C until analysis by flow cytometery (up to 3 d) that was performed on a BD FACS Aria IIu. Data was analyzed by the FlowJo 7.6.5 software (Tree Star Inc, Ashland, OR, USA).

### Polyamine Measurements

In order to measure the polyamine levels of human IECs upon interaction with *Giardia in vitro*, interactions of HCT-8 cells with WB trophozoites were set up and harvested as described for DNA content analysis. Cell pellets were taken up in 500 µl cold PBS and sonicated 2 x 20 seconds. 200 µl of 0.4 M perchloric acid was added to 200 µl of the sonicate and spun 2′ at 20′000 x g, room temperature. Samples were stored at 4°C until further analysis. The residual sonicate was applied to protein concentration determination by Bio-Rad Protein Assay for normalization of polyamine measures. Chromatographic separation and quantitative determination of polyamines were carried out using high performance liquid chromatography (HP 1100) as described in Seiler and Knödgen [17].

### Data Analysis

All data were analyzed using Microsoft Office Excel 2007. Statistical comparison of data was done by student´s t-test (P-values <0.05, significant; <0.01 highly significant).

## Results

### *Giardia* – IEC Interaction: Effects on Arginine Levels in the Medium

First we studied the amino-acid turn-over during *Giardia*-IEC interactions *in vitro*. Caco2 clone TC7 IECs were combined with *Giardia* WB trophozoites (assemblage A). *Giardia* trophozoites interacting with human IECs cells led to an increase of glutamic acid, proline, alanine and ornithine in the interaction culture medium, whereas arginine was drastically reduced already after 1.5 h and no longer detectable after 24 h of interaction (Table 1). Therefore, amino acid levels were also measured at earlier interaction timepoints, after 1 and 2 h (Table S2): The drastic increase of certain amino acids, as well as the fast consumption of arginine, was confirmed. We also studied the effects of trophozoites from assemblage B (GS) and the non-human infecting assemblage E (P15) and these showed the same pattern of amino acid consumption (Table S2). Thus, *Giardia* trophozoites from all three tested assemblages consume arginine quickly during interaction with IECs.

**Table 1:** Amino acid analysis of the medium of the interaction between IECs (Caco2 clone TC7) and *Giardia* trophozoites (isolate WB).

Amino Acid	0 h	1.5 h WB	24 h WB	24 h ctrl
**Aspartic Acid**	1.00	1.20	0.87	0.13
**Threonine**	1.00	1.09	1.11	0.99
**Serine**	1.00	1.01	0.84	0.83
**Glutamic Acid**	1.00	1.39	3.05	0.58
**Proline**	1.00	1.39	1.79	1.68
**Glycine**	1.00	1.09	1.10	0.92
**Alanine**	1.00	1.70	8.07	3.09
**Valine**	1.00	1.10	1.16	0.89
**Methionine**	1.00	1.15	1.13	0.84
**Isoleucine**	1.00	1.08	1.02	0.84
**Leucine**	1.00	1.13	1.05	0.80
**Tyrosine**	1.00	1.07	1.01	0.93
**Phenylalanine**	1.00	1.13	1.05	0.93
**Ornithine**	1.00	6.85	11.49	1.26
**Lysine**	1.00	1.14	1.10	0.92
**Histidine**	1.00	1.11	0.98	0.89
**Arginine**	1.00	0.54	0.00	0.80

Arbitrary units are used.

### Arginine Starvation of IEC

**Figure 1:**
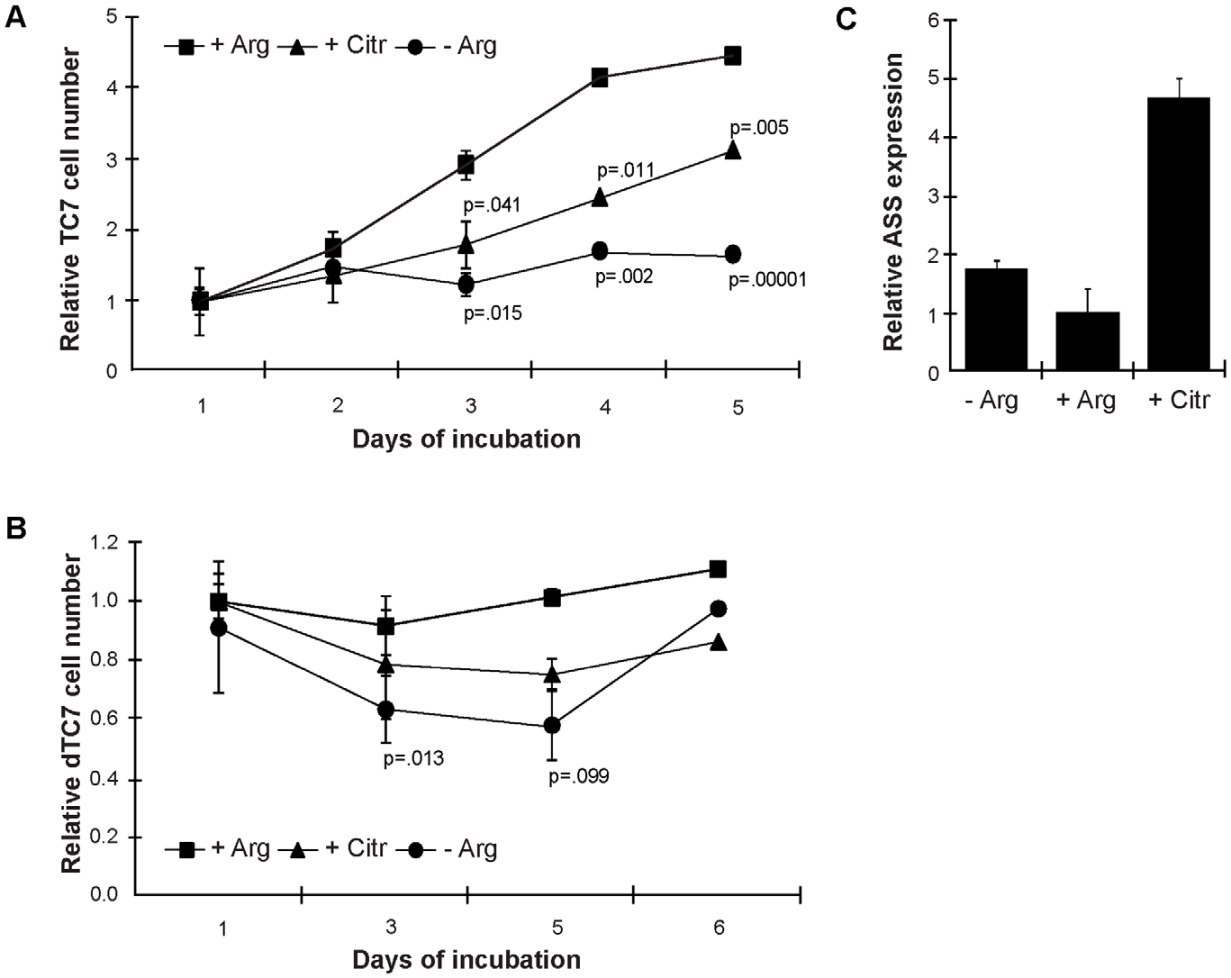
Growth curves of Caco2 cells in arginine-free medium compared to arginine- and citrulline-supplemented medium. Graphs are given for cells growing in complete medium (+Arg, rectangles), in medium without arginine (-Arg, circles) and arginine-free medium complemented with citrulline (+Citr, triangles). In A, cell numbers for proliferating Caco2 cells were measured by MTT assay; in B differentiated Caco2 cells were used. Samples were set up in triplicates, cell numbers expressed as relative to +Arg (d1). P-values calculated in comparison to complete medium values are shown for –Arg and for +Citr. After 3 d, growth was significantly reduced without arginine and citrulline could partially replace for the omitted arginine. In C, argininosuccinate synthase (ASS) mRNA expression in proliferating Caco2 cells at day 2 is displayed for the 3 different growth conditions, as assessed by qPCR in quadruplicates. Note the increase in ASS expression in the presence of citrulline.

To investigate the consequences of arginine starvation on IECs, proliferating TC7 and HCT-8 cells were grown for 5 d in three different media: (i) arginine-free medium, (ii) arginine-free medium supplemented with citrulline or ornithine, the products resulting from the conversion of arginine by the parasite enzymes ADI and OCT, and (iii) complete medium containing arginine. As assessed by the MTT assay, there was a significant reduction of the TC7 cell number after 3 d in arginine-depleted medium compared to complete medium (Figure 1A), increasing with incubation time. Citrulline could partially replace for the omitted arginine in the medium (Figure 1A), which indicates that TC7 cells are capable of converting citrulline into arginine. Ornithine addition to the arginine-free medium did not have any effects (data not shown). HCT-8 cells behaved in the same way as TC7 cells, but citrulline could fully replace for arginine during the first 4 d (Figure S1A). Differentiated, TC7 cells showed fewer differences upon growth in arginine-free medium than proliferating cells (Figure 1B).

To further study the ability of IECs to convert citrulline into arginine, expression of the rate-limiting enzyme, argininosuccinate synthetase (ASS), was assessed. As depicted in Figure 1C, at day 2, TC7 cells up-regulated the expression of ASS (4.67±0.35 times) when citrulline was present in the medium (compared to complete medium). In the absence of arginine, the gene was upregulated slightly (1.76±0.14 times). At all the other time-points, ASS was expressed at basal levels. The same was true for HCT-8 cells, with the difference that the changes were stronger and started already at day 1 (7.21±2.93 for –arginine; 8.36±0.30 times for +citrulline; Figure S1B). Thus, arginine starvation reduces the cell number of IECs and proliferating cells are more sensitive to arginine starvation than differentiated cells. Citrulline can partially restore this arginine starvation-induced effect.

### *Giardia* – IEC Interaction: Effects on Cell Number

**Figure 2:**
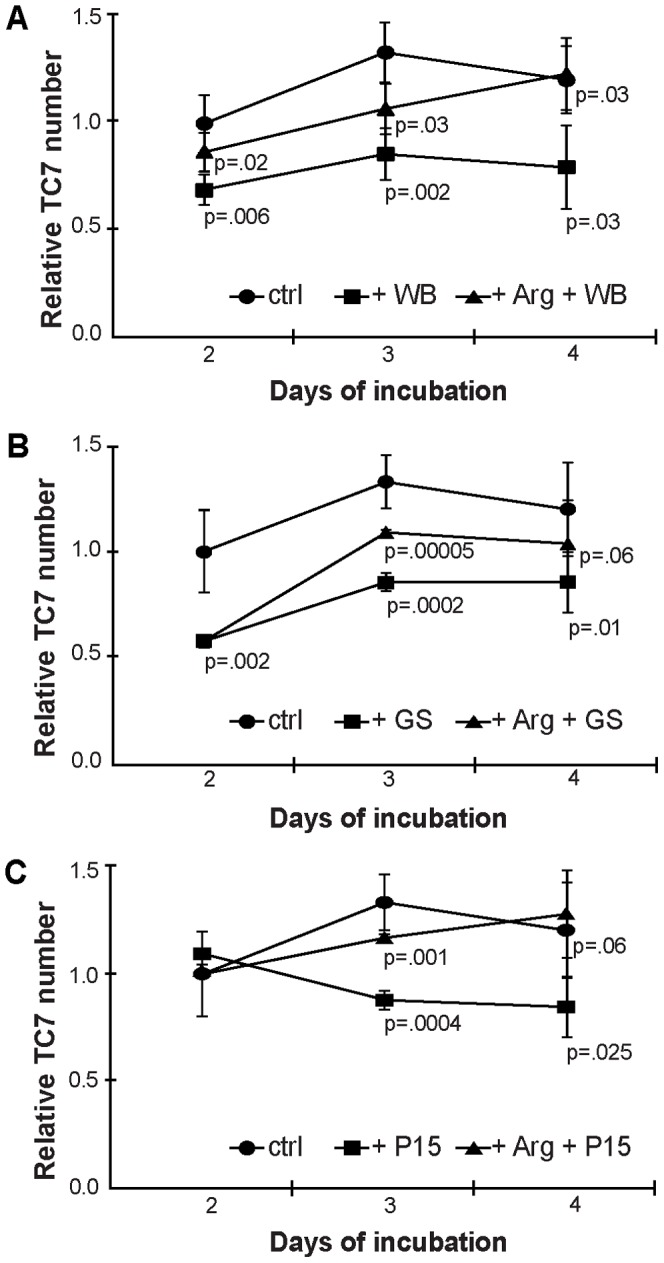
Growth curves of Caco2 cells upon addition of *Giardia* trophozoites and arginine. IEC numbers were monitored by MTT assay over 4 d in triplicates and displayed as relative to ctrl d2. Caco2 cells were challenged with trophozoites of the isolate WB (A), GS (B) or P15 (C). To revert the parasite-induced cell number reduction, arginine was added to 0.4 mM as displayed in the graphs. P-values are given for the differences between control cells (ctrl, circles) and parasite-challenged cells (+WB/GS/P15, rectangles) and between the latter ones and arginine-complemented cells (+Arg +WB/GS/P15, triangles). Note the reduction of Caco2 cell numbers upon parasite addition that could be partially restored by addition of arginine.

Due to the ability of *Giardia* to reduce the amount of arginine in the interaction medium, and thereby locally deplete this amino acid from IECs, the parasite was used as a model pathogen to study arginine starvation. First, the effects on proliferating IECs were analyzed. As assessed by the MTT assay after 2–4 d of interaction between TC7 IECs and WB trophozoites, IEC numbers were significantly reduced compared to medium controls (Figure 2A). L-arginine added to physiological concentrations to the interactions could partially reverse the observed effects (Figure 2A). HCT-8 cells experienced a slighter reduction of cell number in interactions with WB trophozoites already after 2 d, but reached control levels again after 3 and 4 d and addition of L-arginine could partially restore cell numbers (Figure S2). To further expand the analysis of the impact of *Giardia* trophozoites on IECs, trophozoites from the GS and P15 isolates were included in interactions and exhibited comparable effects (Figure 2B, C). This shows that trophozoites from *Giardia* assemblages A, B and E all can reduce the cell numbers of human IECs in an arginine-dependent fashion.

### Arginine Consumption by ADI

**Figure 3:**
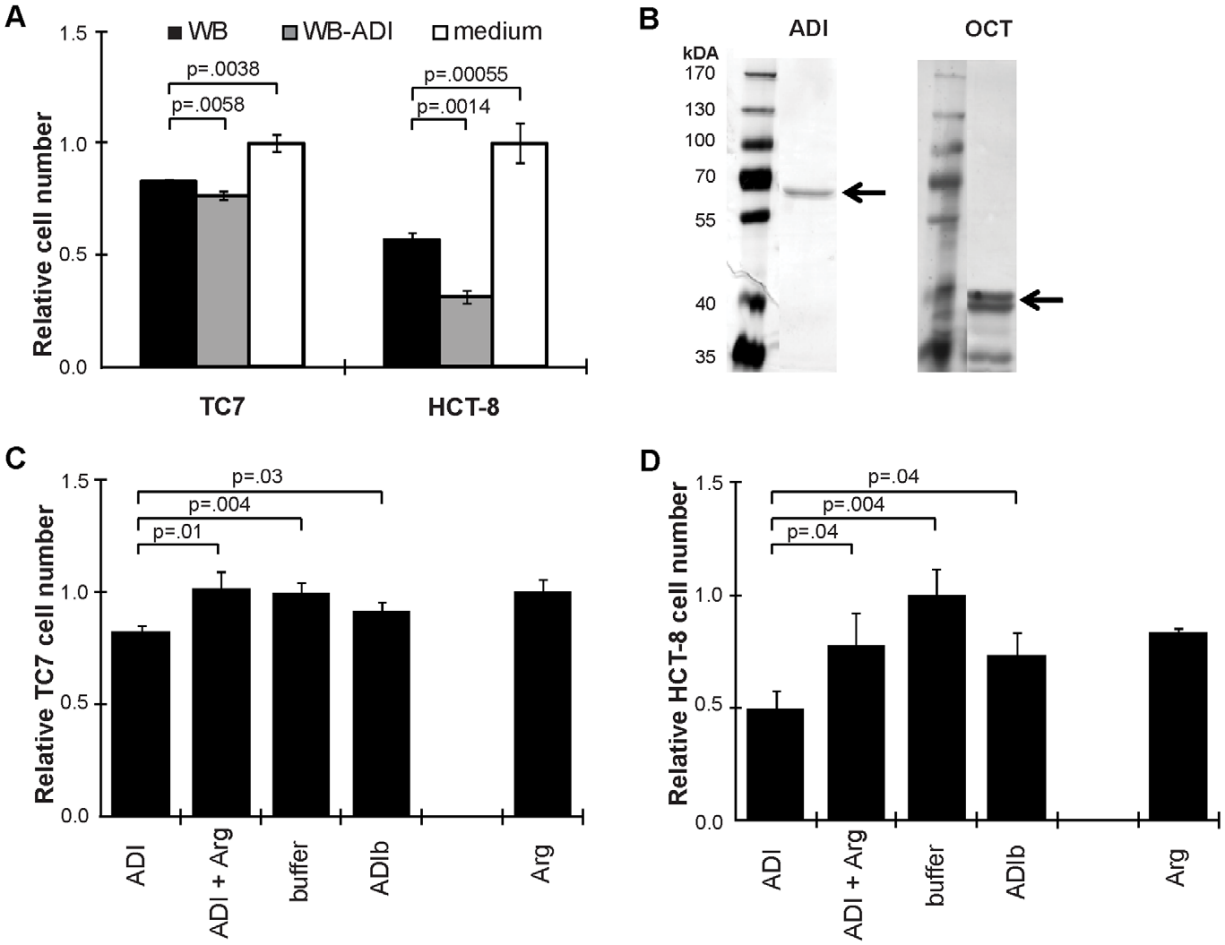
Effects of ADI on IEC numbers. Triplicates of IEC numbers were monitored by MTT assay. In A, IECs (Caco2 and HCT-8 cells), were treated with medium only (medium, white bars), with WB trophozoites (WB, black bars) or with WB trophozoites overexpressing ADI (WB-ADI, grey bars) and measured after 4 d. In B, two silver stained SDS-PAGE gels are shown visualizing the purified ADI and OCT at the expected Mw of 65 kDA and 40 kDA respectively (arrows). In C and D, TC7 and HCT-8 cells were treated with 27.5 U/L ADI from *Giardia* and measured after 4 d (TC7 cells) and 40 h (HCT-8 cells). ADI induced effects were abolished by addition of arginine to 0.4 mM (ADI +Arg). Corresponding controls are given (buffer; ADIb, which is the boiled protein control; Arg that shows that arginine addition alone does not affect the cells). Cell numbers are expressed as relative to buffer ctrl, P-values are displayed. Note the reduction in cell number upon addition of ADI.

Since *Giardia* ADI is the enzyme consuming arginine within the parasite and it is also released increased upon interaction with host cells, special attention was given to this protein. Non-transfected WB trophozoites significantly reduced the IEC number within 4 d, but significantly less than when trophozoites were over-expressing ADI (WB-ADI; Figure 3A). To further assess the role of ADI, and its subsequent enzyme in the arginine dihydrolase pathway, OCT, both proteins were purified from WB trophozoites. Purity and correct size of the proteins (65 kDa and 40 kDa, respectively) was shown by SDS-PAGE and silver staining (Figure 3B). Activities were measured to be 14 U/mg for ADI and 17 U/mg for OCT. Adding ADI to proliferating TC7 cells, thus mimicking the release of ADI by *Giardia* without any other parasite protein or parasites themselves disturbing, led to a highly significantly reduced cell number (compared to buffer control) after 4 and 5 d of incubation (Figure 3C, Figure S3A). The observed effects of ADI and arginine were also seen for HCT-8 cells (Figure 3D), though reaching high significance already much earlier (40 h) (Figure S3B). Addition of OCT did not change this reduction in cell number (Figure S3). Interestingly, the effect observed upon addition of ADI could be reversed by adding L-arginine to the interaction setup (Figure 3 C, D and Figure S3). Thus, *Giardia* is capable of consuming arginine in the surroundings of host cells not only by taking it up, but also by degrading it extracellularly by ADI, and thereby reducing IEC proliferation. ADI added to differentiated TC7 cells had no effect within 5 d of interaction (data not shown). This further confirms that *Giardia* reduces proliferation of IECs via consumption of arginine.

### Effects of Arginine and *Giardia* Infection on IEC Cell Cycle Progression *in vitro*

**Figure 4:**
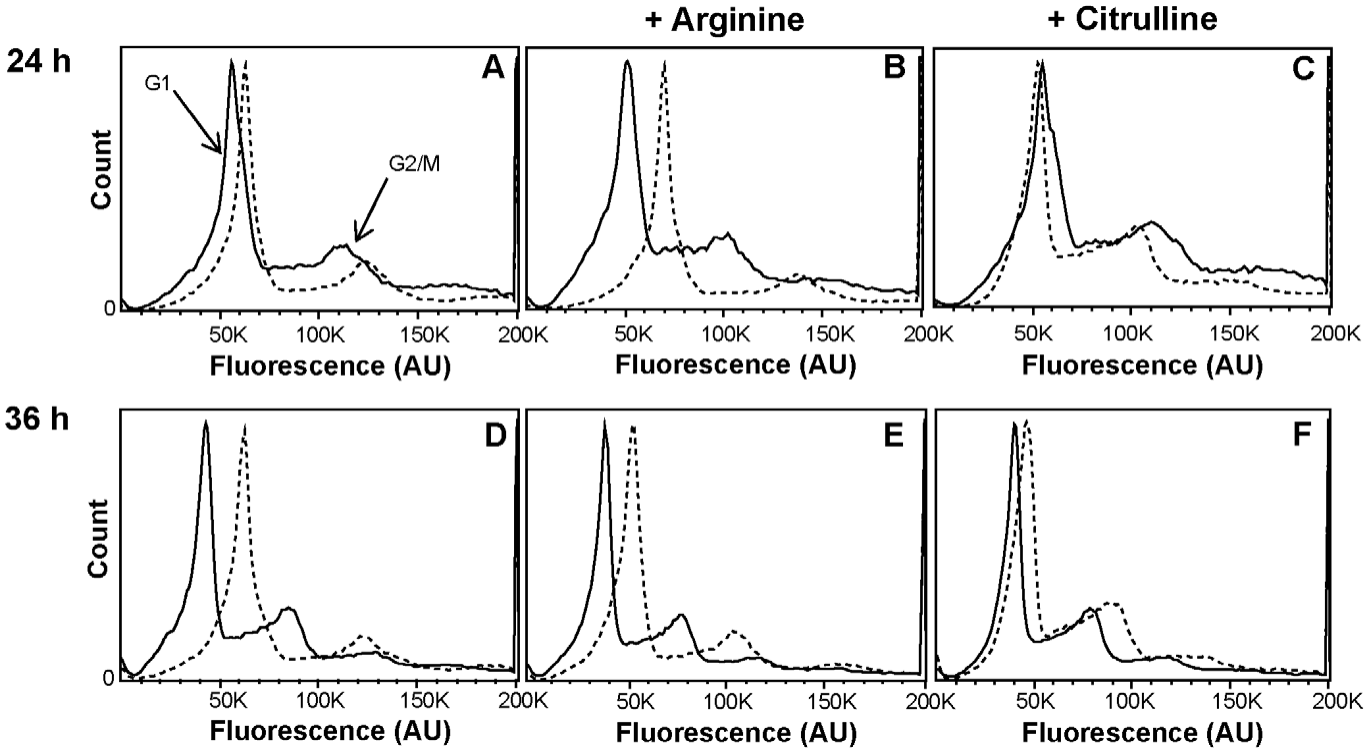
DNA content profiles of HCT-8 cells upon *Giardia*-interaction. The DNA of HCT-8 cells was stained with propidium iodide and analyzed by flow cytometry. The solid lined histograms represent control cells, whereas the dotted lines show profiles of cells after 24 h (A, B and C) and 36 h (D, E and F) of interaction with WB trophozoites. A and D display controls with only water added; in B and E, arginine was added to 0.4 mM; in C and F, citrulline was added to 0.4 mM. Note the histogram shifts upon parasite addition that could be completely reverted by the addition of citrulline.

To further investigate the arginine-dependent reduction in IEC proliferation, we studied the distribution of cells in different parts of the cell cycle by DNA content analysis of HCT-8 cells, grown with and without parasites. Figure 4A shows a reduction of the number of cells in the S-phase and an accumulation mainly in the G1/S transition phase upon IEC-WB interaction. The same effect was observed upon interaction with the two other parasite isolates, GS and P15 (Figure S4). Addition of arginine to these setups could reverse the effects seen upon parasite interaction minimally after 36 h (Figure 4E). Within 24 h, arginine addition rather led to an enhancement of the observed effects (Figure 4B). However, addition of citrulline could fully restore control DNA content profiles (Figure 4C). Thus, parasite-induced arginine starvation leads to cell cycle arrest in IECs, but this can be reduced by the addition of citrulline.

Earlier microarray data suggested that cell cycle inhibitory genes are induced during IEC-*Giardia* interactions [13]. We therefore studied the expression of two of these genes on RNA level; growth arrest and DNA-damage-inducible protein alpha (GADD45A) and B-cell translocation gene 3 (BTG3). BTG3, that is known to induce cell cycle arrest after DNA damage, was down-regulated at the early time-points of interaction in HCT-8 cells (1.5 and 3 h, except for 3 h WB-interaction) and up-regulated after 6 and 24 h with all 3 parasite isolates (Table 2). Expression of GADD45A followed the same profile (Table 2). Similar, but less pronounced, results were obtained in TC7 cells (Table S3). An up-regulation of BTG3 and GADD45A was also observed upon cultivation of IECs in arginine-free medium compared to normal cultivation medium (Table S4).

**Table 2:** mRNA expression of cell cycle regulatory proteins (BTG3, GADD45A) in HCT-8 cells upon parasite interaction.

	BTG3			GADD45A			
	WB			WB			
**0 h**	1.00	±	0.11	1.00	±		0.16
**1.5 h**	0.60	±	0.12	0.55	±		0.06
**3 h**	2.34	±	0.18	2.10	±		0.16
**6 h**	1.62	±	0.17	3.04	±		0.28
**24 h**	3.90	±	0.26	8.66	±		0.84
	**GS**			**GS**			
**0 h**	1.00	±	0.14	1.00	±		0.16
**1.5 h**	0.46	±	0.05	0.53	±		0.05
**3 h**	0.82	±	0.06	0.68	±		0.07
**6 h**	1.34	±	0.20	3.58	±		0.46
**24 h**	6.80	±	0.79	8.59	±		0.46
	**P15**			**P15**			
**0 h**	1.00	±	0.08	1.00	±		0.15
**1.5 h**	0.58	±	0.06	1.02	±		0.37
**3 h**	0.79	±	0.19	1.10	±		0.35
**6 h**	1.34	±	0.10	4.04	±		0.16
**24 h**	6.74	±	0.65	9.60	±		0.99

Expression levels are expressed in arbitrary units.

To further assess the effects of arginine deprivation by *Giardia* on host IECs, we measured polyamine levels in these upon interaction. Spermine was the only polyamine that was clearly reduced upon parasite interaction after 24 h compared to control cells (p = .00011) (Figure S5). Thus co-incubation of *Giardia* trophozoites with IECs induces cell cycle arrest genes and reduces spermine levels, explaining the reduced cell proliferation due to arginine starvation.

## Discussion

**Figure 5:**
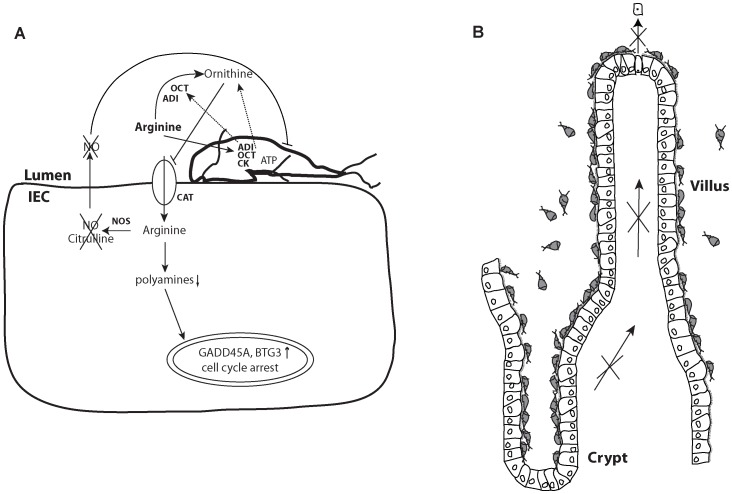
The role of arginine in intestinal infection. An overview of the intestinal epithelial lining is given in B, with a villus and a crypt being depicted. Proliferating cells in the crypts migrate towards the tip of the villus, undergoing differentiation, and are shed at the tip of the villus after cell death. *Giardia* trophozoites sit all along the epithelial surface (with exception of the Paneth cells that are most down in the crypts) [47]. A trophozoite attached to an IEC is shown in A. Dotted lines indicate molelcules released by the trophozoite. Arginine is actively taken up by the parasite and converted into CO_2_, NH_3_ and ornithine along the dihydrolase pathway, indicated by the enzymes ADI (arginine deiminase), OCT (ornithine carbamoyl transferase) and CK (carbamate kinase). Ornithine is released via an arginine-ornithine antiporter, blocks the cationic amino acid transporters (CAT) of IECs and thereby uptake of arginine. In addition, arginine is degraded extracellular by ADI and OCT released from *Giardia* upon interaction. All this leads to reduced arginine availability within the host IEC. As shown within this study, the consequences are reduced polyamine levels in IECs that result in cell cycle arrest via upregulation of cell cycle block genes (GADD45A, BTG3). This reduced cell proliferation in the crypts could lower intestinal cell turnover and reduce cell differentiation along the villus-axis, allowing the parasite to live in a more stable environment. The reduced arginine availability in IECs also leads to decreased production of the antimicrobial agent nitric oxide (NO) via nitric oxide synthases (NOS) in all cells of the intestinal epithelial lining.

In this study, we analyzed the role of arginine in intestinal infection within an *in vitro* model of host-pathogen interaction: human IECs and the protozoan parasite *Giardia intestinalis*. The use of proliferative, non-differentiated Caco2 and HCT-8 cells hereby can be regarded as a simplified model for somatic stem cells lying in the crypts of the intestinal epithelium. They are actively dividing, migrate towards the top of the villi, give rise to the intestinal epithelium and are important for maintenance of the epithelium´s integrity (Figure 5) [18]. Differentiated Caco2 cells were used as a model for mature epithelial cells being situated in the villi. They resorb nutrients and act as a physical barrier [18]. Spontaneous *in vitro* differentiation of Caco2 cells to illeal-like enterocytes upon cultivation for 21 d was used for this study [13], [19].

It has been shown that *Giardia* trophozoites are able to reduce the amount of arginine in their own growth medium [20]. We could complement these findings with proving that the same holds true in an interaction setup with human IECs and 3 isolates of *Giardia,* representing 3 different assemblages (Table 1, Table S2). We focused on the effects of *Giardia* on IECs and found that it reduces cell numbers of proliferating Caco2 and HCT-8 cells in an arginine-dependent manner (Figure 2, Figure S2). With prolonged incubation, cells were able to recover and reach control levels again. This shows clearly the limitations of an *in vitro* based interaction system, where only a small portion of parasites survives longer than 24 h and effects on host cells are visible only within a certain timeframe. However, it is only possible within an *in vitro* setup to study the very first hours of intestinal infection.

To assess the role of arginine on growth of IECs *in vitro* more generally, human IECs were cultivated in medium without arginine (Figure 1, Figure S1). With increasing incubation time, proliferation of IECs was reduced. Citrulline could substitute for arginine in proliferating IECs, whereas ornithine could not. In differentiated IECs, citrulline could not replace arginine. This was also in accordance with expression of ASS that increased within one day of culture in arginine-free medium and even more in citrulline-containing medium in proliferating IECs, but not in differentiated IECs. *In vivo*, high expression of ASS and ASL in IECs was shown in children up to 3 years, probably because there is not enough arginine available in mother milk [21], [22]. The expression status of ASS in intestines of adult individuals has not been assessed so far.

Interestingly, *Giardia* takes up arginine by an arginine-ornithine antiporter [23]. Thereby ornithine is released to the close surrounding of the parasite (Figure 5). Ornithine inhibits arginine uptake by IECs [24], but not by *Giardia*. Hereby the parasite is able to starve host IECs even more from arginine. In addition, it has been shown that the parasite releases enzymes upon host-interaction that are involved in arginine metabolism: ADI and OCT (Figure 5) [10]. Addition of trophozoites over-expressing ADI, or of purified ADI, to proliferating IECs led to a decrease in cell proliferation that could be restored by addition of arginine (Figure 3). As expected, effects of ADI addition were not as strong as growth of cells in arginine-free medium, because IECs are able to reconvert citrulline into arginine upon induction of ASS. The addition of OCT in combination with ADI could not increase the reduction in cell proliferation, because the reverse reaction catalyzed by OCT (ornithine to citrulline), is 12 × faster than the forward reaction [9]. Also did the addition of pure ADI not lead to as strong effects as of ADI-transfectants, since these parasites were additionally consuming arginine. ADI had no effect on differentiated IECs, indicating that active cell proliferation was reduced through the parasités arginine-consumption rather than inducing apoptosis. These data highlight the importance of ADI for *Giardia* and offer this parasite protein as a new possible drug or vaccine target for treatment of giardiasis.

Induction of host cell apoptosis by *Giardia* trophozoites has been described to take place *in vitro* [25], [26] as well as, though to a subtle extent, *in vivo* [27] and was therefore described as one of the mechanisms leading to epithelial dysfunction in giardiasis [28]. Within our *in vitro* setup, we could not confirm the early induction of caspase-3 activation in IECs upon parasite-interaction (data not shown). Possibly, exposure to higher ratios of parasites and longer incubation times would exert effects of *Giardia* trophozoites on apoptosis. It might also be possible that the described increase in caspase-3 activation is dependent on host cell line and *Giardia* isolate used. Growth of IECs in medium without arginine did not increase their intrinsic apoptotic rates either (data not shown). However, we have not further investigated other, caspase-independent ways of apoptosis, such as for example autophagic cell death that was shown to result from arginine-withdrawal upon ADI-treatment of tumor cells ([29], [30]).

Further, the parasite’s arginine-consuming effects were assessed by flow cytometry: The DNA content profiles of human IECs (Figure 4, Figure S4) show a clear shift upon IEC interaction with parasites. This mostly can be seen as the IEĆs cell cycle being blocked in G1/S transition and S phase being reduced. Within 24 h of incubation, addition of arginine enhanced this effect, probably due to additional feeding of parasites with arginine. At 36 h, however, addition of arginine had a positive effect also on IECs, leading to a slight back-shift of the DNA content profile. Interestingly, the arginine metabolite citrulline could fully restore control DNA content profiles in parasite-interacting IECs after 24 and 36 h. Citrulline can, as discussed above, be reconverted into arginine by the IECs used in this study. However, this does not hold true for *Giardia* parasites that use arginine solely as a substrate for the arginine dihydrolase pathway [9]. These findings are interesting with regards to oral rehydration therapy (ORT) that is used as general treatment of infectious diarrhea [31]. Arginine and citrulline are included in certain ORT formulations, but it is not clear what beneficial effects they have. Supplementation with arginine is not recommended, because it can cause excessive urea production in the liver [21] and at high concentrations even diarrhea [32] and in the case of giardiasis, is expected to support the growth of parasites. Therefore, further focus should be put on citrulline as an addition in ORT or as a nutritional supplement against intestinal infections. Further studies are needed to determine the best ways and concentration of citrulline supplementation before implementation in endemic regions will be feasible. As mentioned earlier in the discussion, it needs to be assessed though, whether the two crucial enzymes for citrulline reconversion into arginine, ASS and ASL, are expressed in intestines of arginine-deprived organisms. A recent *in vivo* study in mice, however, shows that citrulline supplementation leads to increased arginine-levels in jejunal tissue [33]. Further, apart from direct uptake of external arginine or citrulline reconversion into arginine, also lysosomal and proteosomal protein breakdown might be an additional source of arginine for host cells [34]. Future *in vivo* studies will show whether treatment with citrulline could reduce disease symptoms of intestinal infections and help the immune system of patients to clear infections more efficiently. Compared to other therapeutic control measurements for giardiasis, lower costs, higher practicability and beneficial side effects on other coinfecting pathogens, such as *e.g. Helicobacter pylori* [3], could supposedly be big advantages of citrulline supplementation.

To figure out why arginine consumption by *Giardia* led to a halt in cell proliferation, we measured polyamine levels upon parasite-interaction (Figure S5). Polyamines are important in cell-cycle progression, their levels doubling during a cell cycle [35]. Polyamines can be either taken up by cells or synthesized from arginine. Hereby arginine is converted into ornithine by the action of arginases, and then further into putrescine by ornithine decarboxylase (ODC). Spermidine and spermine are formed by spermidine and spermine synthase, respectively, the three polyamines being interconvertible [35]. Thus, polyamine synthesis is dependent on the availability of arginine. Crucial are also ODC levels that we found to be upregulated 9.5 × after 24 h of parasite-interaction on RNA level (data not shown), implying that cells responded to the polyamine depletion. We observed a highly significant reduction of spermine levels in parasite-interactions compared to controls. Thus, through arginine consumption, *Giardia* reduces polyamine levels and thereby leads to a halt in cell cycle.

To maintain epithelial integrity and homeostasis, a balance between epithelial cell proliferation, differentiation, migration and death is needed [12]. Intestinal pathogens are known to affect this homeostasis in various specific ways in order to slow down the rapid epithelial cell turnover and maintain the intestinal epithelium as a niche for colonization [12]. As suggested by our study, intestinal epithelial proliferation could also be reduced in a more general way through arginine consumption by intestinal pathogens such as *Giardia* and thereby favoring the colonization of the epithelium even though precise regulatory mechanisms for parasite detachment and reattachment *in vivo* need to be studied in future. The advantage for the parasite with this arginine-consumption approach is that also a part of the innate immune response, NO production, is reduced at the same time. We observed a reduced proliferation of IECs *in vitro* in short term upon local consumption of arginine by *Giardia*. In long term co-cultures this is expected to lead to caspase-independent apoptosis [36], [37], [38]. We therefore propose that *in vivo Giardia* could reduce proliferation of intestinal stem cells in the crypts via local consumption of arginine and thereby affect intestinal tissue homeostasis. Consequently, constantly lost cells at the top of the villi of the intestinal epithelium would not be adequately replaced (Figure 5). Thus, we speculate that arginine deprivation could, via induction of apoptosis in IECs, in the longer term lead to villus shortening, increased intestinal permeability and diarrhea [39]. Villus shortening and reduction of the intestinal surface has been observed *in vivo* before [36], [37], [38], [39], [40]. Increased intestinal permeability due to apoptosis has been described previously to be a reason for diarrhea in giardiasis [28], [41]. It is important to note that giardiasis is a multifactorial disease that is characterized by involving contributions from both, host and parasite, as reviewed by Cotton et al [41]: The pathophysiology of giardiasis includes lowered intestinal barrier function by the above-mentioned caspase-dependent IEC apoptosis [26], [27], [42] and reduction or redistribution of cytoskeleton proteins [43]. Further, anion hypersecretion [44], increased intestinal transit rates due to mast cell degranulation [45], brush border shortening due to activation of lymphocytes [40], maldigestion due to brush border enzyme deficiencies [28] and small intestinal malabsorption [36] are causes of diarrhea in giardiasis [41], [46]. The here described arginine-dependent effects add another bit to the understanding of the multifactorial disease giardiasis by directly linking parasite factors to effects observed in host cells and offer new possibilities for future ways of treatment.

Taken together, the 2 different intestinal cell lines used in these *in vitro* interaction studies exhibited slightly different responses to arginine-deprivation time-wise, but followed the same principles: they exhibited a reduced proliferation upon incubation with *Giardia* trophozoites, with ADI, or in arginine-free medium and addition of citrulline and arginine could restore growth fully and partially respectively. Proliferation stop was associated with an up-regulation of cell cycle inhibitory genes and could, through the arginine-deprivation by parasites, be a result of reduced polyamine synthesis. This reduced epithelial turnover could favor host colonization *in vivo* and might, if continued, lead to epithelial apoptosis and thereby increased intestinal permeability and diarrhea.

## Supporting Information

Figure S1 **Growth curves of Caco2 cells in arginine-free medium compared to arginine- and citrulline-complemented medium.** Graphs are given for cells growing in complete medium (+Arg, rectangles), in medium without arginine (-Arg, circles) and arginine-free medium complemented with citrulline (+Citr, triangles). In A, cell numbers for proliferating HCT-8 cells were measured by MTT assay. Samples were set up in triplicates. Cell numbers were normalized to complete medium values at day 1. P-values calculated in comparison to complete medium values are shown for –Arg and for +Citr. After 2 d, growth was significantly reduced without arginine and citrulline could replace for the omitted arginine. In B, argininosuccinate synthase (ASS) mRNA expression in HCT-8 cells at day 2 is displayed for the 3 different growth conditions, as assessed by qPCR. Note the increase in ASS expression in the presence of citrulline.
(TIF)

Figure S2 **Growth curves of HCT-8 cells upon addition of** ***Giardia*** **trophozoites and arginine.** Intestinal epithelial cell numbers were monitored by MTT assay over 4 d in triplicates. Results for days 2–4 were normalized to d1 control cell values and are displayed. HCT-8 cells were challenged with trophozoites of the isolate WB. To revert the parasite-induced cell number reduction, arginine was added to 0.4 mM as also displayed in the graph (+Arg +WB, triangles). P-value is given for the difference between control cells (ctrl, circles) and parasite-challenged cells (+WB, rectangles). At day 2, HCT-8 cell numbers clearly reduced upon parasite addition and this could be partially restored by addition of arginine.
(TIF)

Figure S3 **The effects of ADI and OCT on intestinal epithelial cell numbers.** Triplicates of intestinal epithelial cell numbers were monitored by MTT assay during 120 h for Caco2 cells (clone TC7, A) and during 46 h for HCT-8 cells (B). IECs were treated with 27.5 U/L ADI and 43.5 U/L OCT produced in and purified from *Giardia* trophozoites. Effects were abolished by addition of arginine to 0.4 mM (ADI +Arg and ADI +OCT +Arg). Corresponding controls are given (ADIb, ADIb +OCTb, buffer ADI, buffer ADI OCT, Arg that shows that arginine addition alone does not affect the cells). Note the reduction in cell number upon addition of ADI.
(TIF)

Figure S4 **DNA content profiles of HCT-8 cells upon** ***Giardia*** **-interaction.** The DNA of HCT-8 cells was stained with propidium iodide and measured by flow cytometry. The solid lined histogram represents control cells, whereas the dotted lines show the profiles of HCT-8 cells after 24 h of interaction with WB (A), GS (B) and P15 (C) trophozoites respectively. Note the histogram shifts upon parasite interaction.
(TIF)

Figure S5 **Polyamine levels in HCT-8 cells upon** ***Giardia*** **-interaction.** Samples of interactions were taken after 1.5, 6 and 24 h in triplicates. Levels of the polyamines putrescine, spermidine and spermine in HCT-8 cell extracts were analyzed by HPLC and expressed as nmol per total protein amount in mg. Highly significant different spermine concentrations are visible after 24 h of interaction.
(TIF)

Table S1 **List of primers used for qPCR.**
(DOCX)

Table S2 **Amino acid analysis of medium of the interaction between IEC (Caco2 clone TC7) and Giardia trophozoites (isolates WB, GS, P15).** Arbitrary units are used.
(DOCX)

Table S3 **mRNA expression of cell cycle regulatory proteins (BTG3, GADD45A) in TC7 cells upon parasite interaction.** Expression levels are expressed in arbitrary units.
(DOCX)

Table S4 **mRNA expression of cell cycle regulatory proteins (BTG3, GADD45A) in undifferentiated TC7 and HCT-8 cells upon growth in RPMI with and without arginine (+arg, -arg).** Expression levels are expressed in arbitrary units.
(DOCX)
