# Supplementary figures and images for: Arginine Consumption by the Intestinal Parasite Giardia intestinalis Reduces Proliferation of Intestinal Epithelial Cells
Source: PLoS One. 2012 Sep 19;7(9):e45325. doi: 10.1371/journal.pone.0045325 (PMC3446895; doi:10.1371/journal.pone.0045325)

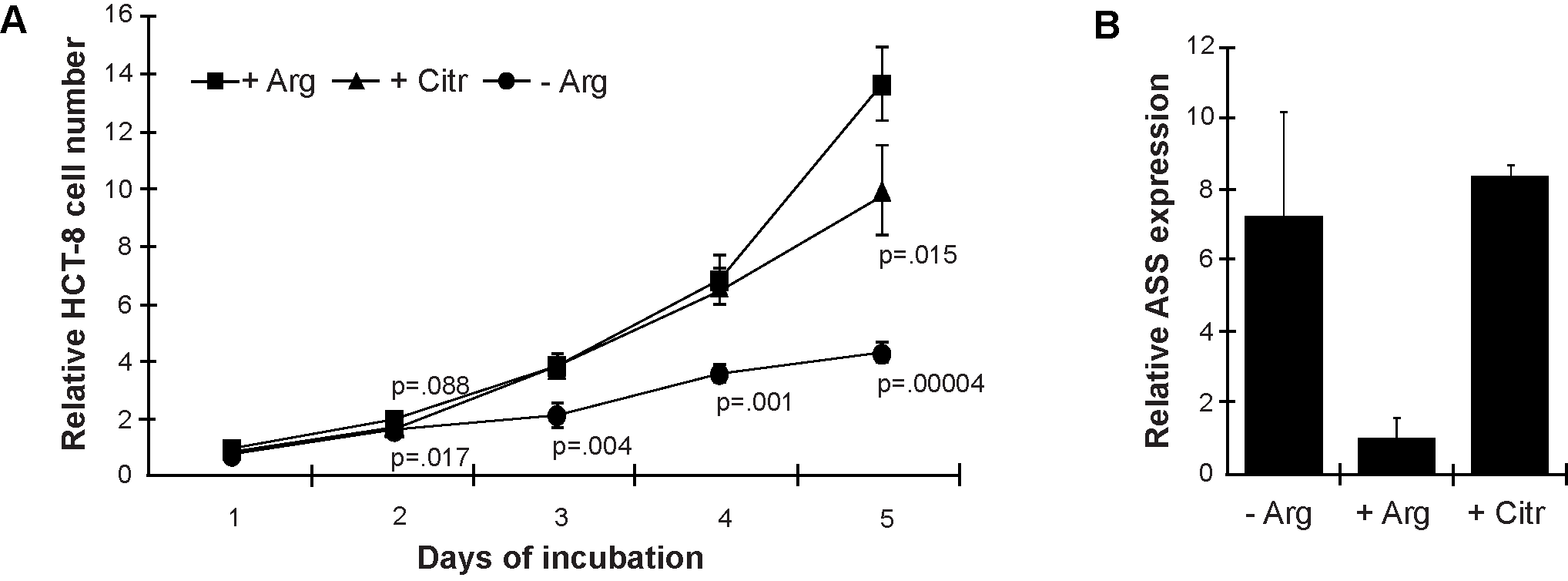

Supplement: Figure S1 — Growth curves of Caco2 cells in arginine-free medium compared to arginine- and citrulline-complemented medium. Graphs are given for cells growing in complete medium (+Arg, rectangles), in medium without arginine (-Arg, circles) and arginine-free medium complemented with citrulline (+Citr, triangles). In A, cell numbers for proliferating HCT-8 cells were measured by MTT assay. Samples were set up in triplicates. Cell numbers were normalized to complete medium values at day 1. P-values calculated in comparison to complete medium values are shown for –Arg and for +Citr. After 2 d, growth was significantly reduced without arginine and citrulline could replace for the omitted arginine. In B, argininosuccinate synthase (ASS) mRNA expression in HCT-8 cells at day 2 is displayed for the 3 different growth conditions, as assessed by qPCR. Note the increase in ASS expression in the presence of citrulline. (TIF) [file pone.0045325.s001.tif]

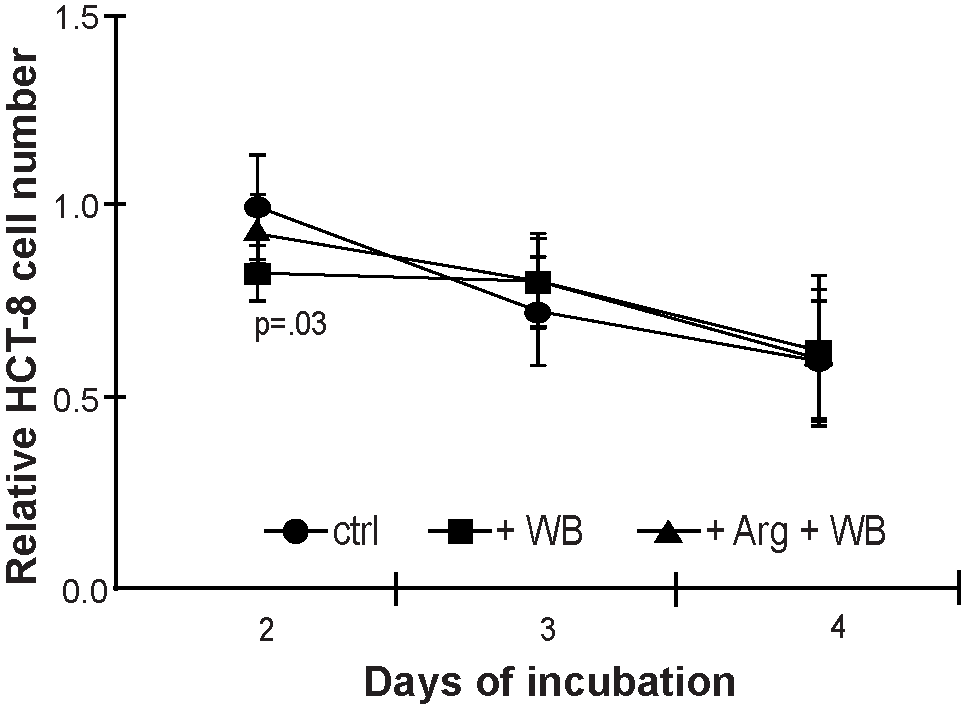

Supplement: Figure S2 — Growth curves of HCT-8 cells upon addition of Giardia trophozoites and arginine. Intestinal epithelial cell numbers were monitored by MTT assay over 4 d in triplicates. Results for days 2–4 were normalized to d1 control cell values and are displayed. HCT-8 cells were challenged with trophozoites of the isolate WB. To revert the parasite-induced cell number reduction, arginine was added to 0.4 mM as also displayed in the graph (+Arg +WB, triangles). P-value is given for the difference between control cells (ctrl, circles) and parasite-challenged cells (+WB, rectangles). At day 2, HCT-8 cell numbers clearly reduced upon parasite addition and this could be partially restored by addition of arginine. (TIF) [file pone.0045325.s002.tif]

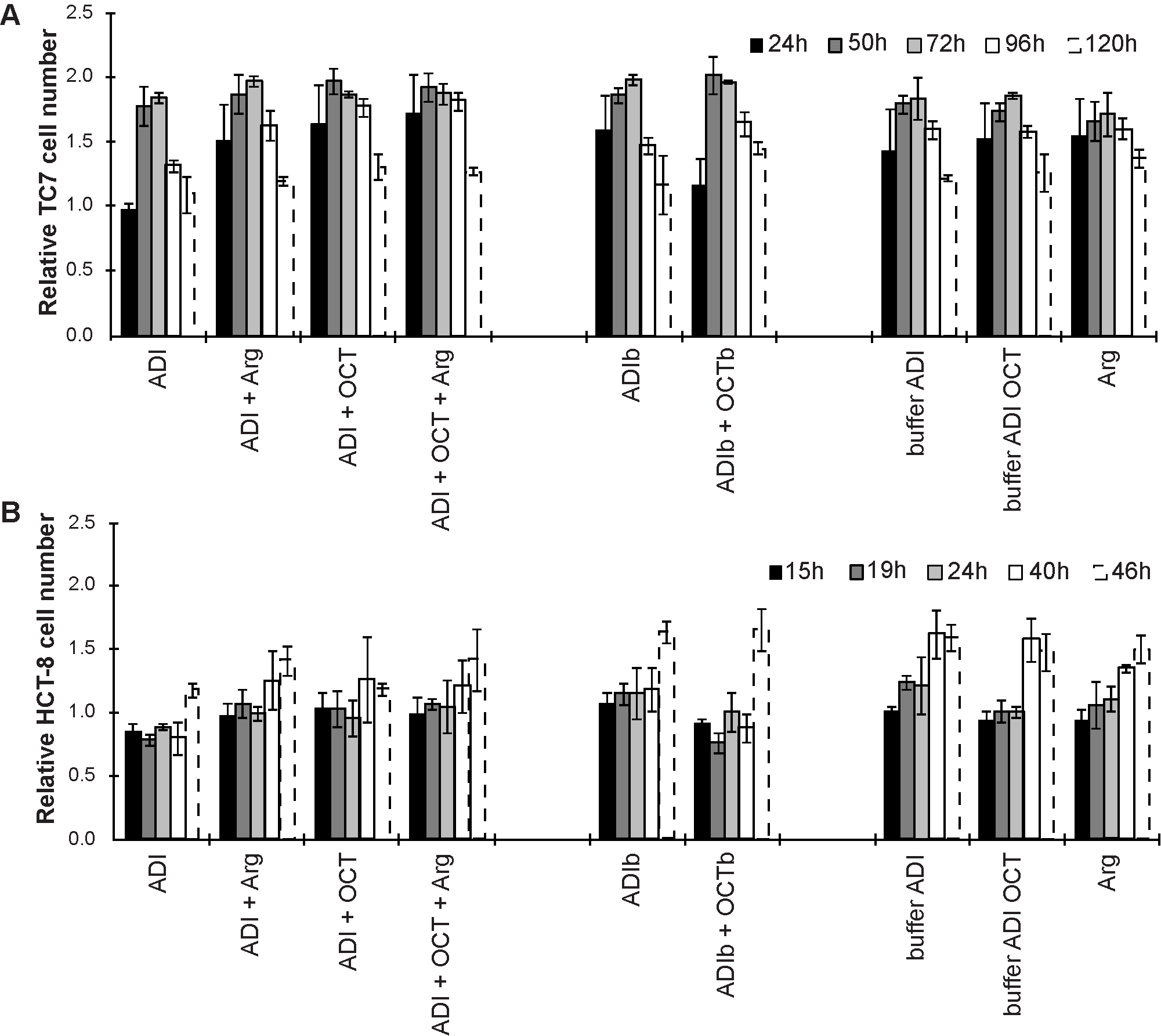

Supplement: Figure S3 — The effects of ADI and OCT on intestinal epithelial cell numbers. Triplicates of intestinal epithelial cell numbers were monitored by MTT assay during 120 h for Caco2 cells (clone TC7, A) and during 46 h for HCT-8 cells (B). IECs were treated with 27.5 U/L ADI and 43.5 U/L OCT produced in and purified from Giardia trophozoites. Effects were abolished by addition of arginine to 0.4 mM (ADI +Arg and ADI +OCT +Arg). Corresponding controls are given (ADIb, ADIb +OCTb, buffer ADI, buffer ADI OCT, Arg that shows that arginine addition alone does not affect the cells). Note the reduction in cell number upon addition of ADI. (TIF) [file pone.0045325.s003.tif]

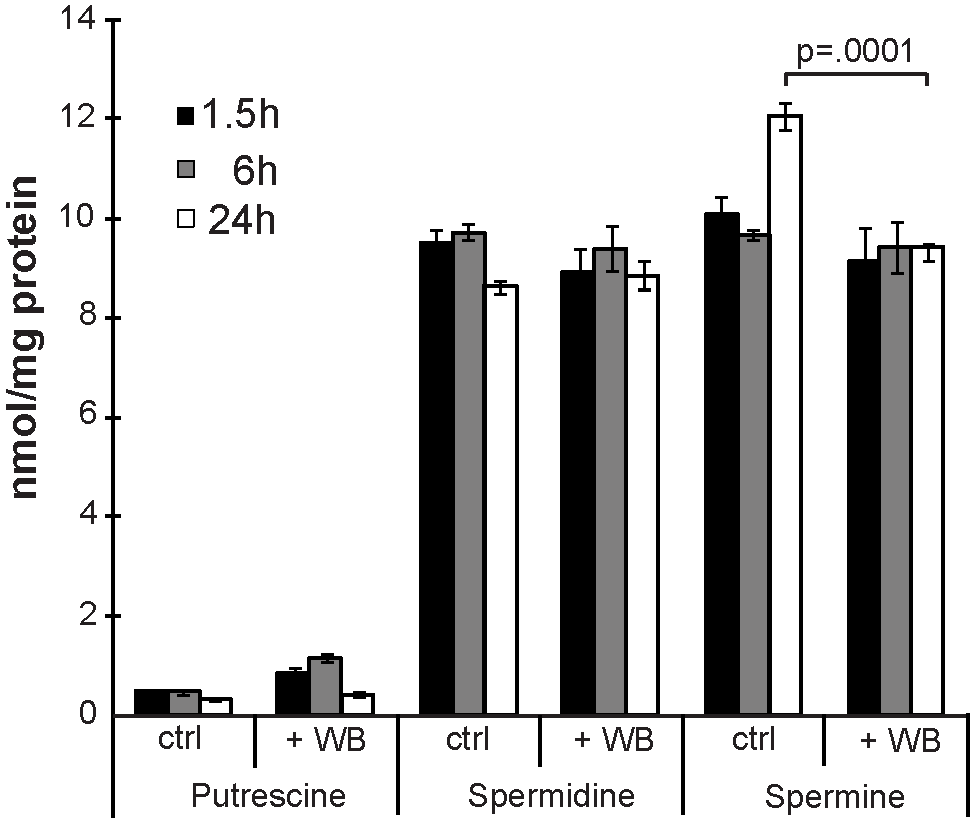

Supplement: Figure S5 — Polyamine levels in HCT-8 cells upon Giardia -interaction. Samples of interactions were taken after 1.5, 6 and 24 h in triplicates. Levels of the polyamines putrescine, spermidine and spermine in HCT-8 cell extracts were analyzed by HPLC and expressed as nmol per total protein amount in mg. Highly significant different spermine concentrations are visible after 24 h of interaction. (TIF) [file pone.0045325.s005.tif]
