# Supplementary material for: Arginine Consumption by the Intestinal Parasite Giardia intestinalis Reduces Proliferation of Intestinal Epithelial Cells
Source: PLoS One. 2012 Sep 19;7(9):e45325. doi: 10.1371/journal.pone.0045325 (PMC3446895; doi:10.1371/journal.pone.0045325)
Supplement: Table S1 — List of primers used for qPCR. (DOCX) [file pone.0045325.s006.docx]

**Table S1.** List of primers used for qPCR.

| **Gene name** | **Accesion nb.** | **abbreviation** | **Primer** |
| --- | --- | --- | --- |
| Argininosuccinate sunthetase 1 (ASS1) | [X01630](http://www.ebi.ac.uk/ena/data/view/X01630) | ASS-F2 | AGCCAAAGCCCCCAACAC |
|  |  | ASS-R2 | GGACCCCTTTTTTGAACTCG |
| Glyceraldehyde 3-phosphate dehydrogenase (GAPDH) | [X01677](http://www.ebi.ac.uk/ena/data/view/X01677) | GAPDH-F2 | AATCCCATCACCATCTTCCAG |
|  |  | GAPDH-R2 | CAGCATCGCCCCACTTG |
| Growth arrest and DNA damage inducible protein 45 A (GADD45A) | P24522 | GADD45A_F | CGAGGACGACGACAGAGA |
|  |  | GADD45A_R | AGCAAAACGCCTGGAT |
| B-cell translocation gene (BTG3) | [BT007276](http://www.ebi.ac.uk/ena/data/view/BT007276) | BTG3_F | TGTGAGGTGTGCTGTCGTAGAGA |
|  |  | BTG3_R | AGGTCAGGAGTTTGAGAGCAGTC |
