# Supplementary material for: Arginine Consumption by the Intestinal Parasite Giardia intestinalis Reduces Proliferation of Intestinal Epithelial Cells
Source: PLoS One. 2012 Sep 19;7(9):e45325. doi: 10.1371/journal.pone.0045325 (PMC3446895; doi:10.1371/journal.pone.0045325)
Supplement: Table S2 — Amino acid analysis of medium of the interaction between IEC (Caco2 clone TC7) and Giardia trophozoites (isolates WB, GS, P15). Arbitrary units are used. (DOCX) [file pone.0045325.s007.docx]

**Table S2.** Amino acid analysis of medium of the interaction between IEC (Caco2 clone TC7) and *Giardia* trophozoites (isolates WB, GS, P15). Arbitrary units are used.

|  |  |  |  |  |  |  |  |  |
| --- | --- | --- | --- | --- | --- | --- | --- | --- |
| **Component** | **0h** | **1h WB** | **1h GS** | **1h P15** | **2h WB** | **2h GS** | **2h P15** | **24h ctrl** |
| **Aspartic Acid** | 1.00 | 1.14 | 1.53 | 1.21 | 1.13 | 1.56 | 1.26 | 0.13 |
| **Threonine** | 1.00 | 1.06 | 1.13 | 1.06 | 1.08 | 1.12 | 1.10 | 0.99 |
| **Serine** | 1.00 | 1.27 | 1.33 | 1.14 | 1.15 | 1.33 | 1.13 | 0.83 |
| **Glutamic Acid** | 1.00 | 1.23 | 1.70 | 1.33 | 1.30 | 1.79 | 1.48 | 0.58 |
| **Proline** | 1.00 | 1.27 | 1.74 | 1.20 | 1.44 | 2.09 | 1.64 | 1.68 |
| **Glycine** | 1.00 | 1.06 | 1.13 | 1.06 | 1.05 | 1.16 | 1.08 | 0.92 |
| **Alanine** | 1.00 | 1.43 | 1.99 | 1.55 | 1.53 | 2.23 | 1.83 | 3.09 |
| **Valine** | 1.00 | 1.04 | 1.14 | 1.05 | 1.05 | 1.18 | 1.08 | 0.89 |
| **Methionine** | 1.00 | 1.09 | 1.22 | 1.09 | 1.08 | 1.30 | 1.13 | 0.84 |
| **Isoleucine** | 1.00 | 1.04 | 1.12 | 1.04 | 1.04 | 1.14 | 1.06 | 0.84 |
| **Leucine** | 1.00 | 1.10 | 1.35 | 1.14 | 1.10 | 1.37 | 1.16 | 0.80 |
| **Tyrosine** | 1.00 | 1.05 | 1.07 | 1.03 | 1.05 | 1.09 | 1.06 | 0.93 |
| **Phenylalanine** | 1.00 | 1.11 | 1.33 | 1.14 | 1.11 | 1.34 | 1.15 | 0.93 |
| **Ornithine** | 1.00 | 6.38 | 8.24 | 8.19 | 10.19 | 11.75 | 12.77 | 1.26 |
| **Lysine** | 1.00 | 1.11 | 1.31 | 1.14 | 1.12 | 1.34 | 1.18 | 0.92 |
| **Histidine** | 1.00 | 1.08 | 1.24 | 1.09 | 1.10 | 1.24 | 1.13 | 0.89 |
| **Arginine** | 1.00 | 0.99 | 0.73 | 0.56 | 0.44 | 0.48 | 0.27 | 0.80 |
