# Supplementary material for: Arginine Consumption by the Intestinal Parasite Giardia intestinalis Reduces Proliferation of Intestinal Epithelial Cells
Source: PLoS One. 2012 Sep 19;7(9):e45325. doi: 10.1371/journal.pone.0045325 (PMC3446895; doi:10.1371/journal.pone.0045325)
Supplement: Table S3 — mRNA expression of cell cycle regulatory proteins (BTG3, GADD45A) in TC7 cells upon parasite interaction. Expression levels are expressed in arbitrary units. (DOCX) [file pone.0045325.s008.docx]

**Table S3.** mRNA expression of cell cycle regulatory proteins (BTG3, GADD45A) in TC7 cells upon parasite interaction. Expression levels are expressed in arbitrary units.

|  | **BTG3** | | | **GADD45A** | | | | |
| --- | --- | --- | --- | --- | --- | --- | --- | --- |
|  | **WB** |  |  | **WB** | | |  |  |
| **0h** | 1.00 | ± | 0.05 | 1.00 | ± | 0.26 | | |
| **1.5h** | 0.40 | ± | 0.04 | 1.20 | ± | 0.06 | | |
| **3h** | 0.51 | ± | 0.06 | 1.01 | ± | 0.35 | | |
| **6h** | 1.50 | ± | 0.21 | 0.71 | ± | 0.08 | | |
| **24h** | 2.28 | ± | 0.39 | 0.77 | ± | 0.05 | | |
|  |  |  |  |  |  |  | | |
|  | **GS** |  |  | **GS** |  |  | | |
| **0h** | 1.00 | ± | 0.08 | 1.00 | ± | 0.26 | | |
| **1.5h** | 0.65 | ± | 0.08 | 3.12 | ± | 0.15 | | |
| **3h** | 0.65 | ± | 0.03 | 2.56 | ± | 0.46 | | |
| **6h** | 1.27 | ± | 0.15 | 0.50 | ± | 0.04 | | |
| **24h** | 2.49 | ± | 0.39 | 0.62 | ± | 0.04 | | |
|  |  |  |  |  |  |  | | |
|  | **P15** |  |  | **P15** |  |  | | |
| **0h** | 1.00 | ± | 0.17 | 1.00 | ± | 0.07 | | |
| **1.5h** | 0.77 | ± | 0.11 | 1.68 | ± | 0.12 | | |
| **3h** | 0.51 | ± | 0.02 | 1.11 | ± | 0.10 | | |
| **6h** | 1.28 | ± | 0.14 | 0.57 | ± | 0.09 | | |
| **24h** | 3.24 | ± | 0.46 | 1.04 | ± | 0.12 | | |
