# Supplementary material for: Arginine Consumption by the Intestinal Parasite Giardia intestinalis Reduces Proliferation of Intestinal Epithelial Cells
Source: PLoS One. 2012 Sep 19;7(9):e45325. doi: 10.1371/journal.pone.0045325 (PMC3446895; doi:10.1371/journal.pone.0045325)
Supplement: Table S4 — mRNA expression of cell cycle regulatory proteins (BTG3, GADD45A) in undifferentiated TC7 and HCT-8 cells upon growth in RPMI with and without arginine (+arg, -arg). Expression levels are expressed in arbitrary units. (DOCX) [file pone.0045325.s009.docx]

**Table S4.** mRNA expression of cell cycle regulatory proteins (BTG3, GADD45A) in undifferentiated TC7 and HCT-8 cells upon growth in RPMI with and without arginine (+arg, -arg). Expression levels are expressed in arbitrary units.

| **+arg** | **BTG3** |  |  | **GADD45A** | | |  | **BTG3** |  |  | **GADD45A** | | |
| --- | --- | --- | --- | --- | --- | --- | --- | --- | --- | --- | --- | --- | --- |
| **0h** | 1.00 | ± | 0.08 | 1.00 | ± | 2.82 |  | 1.00 | ± | 0.07 | 1.00 | ± | 0.21 |
| **1.5h** | 0.61 | ± | 0.03 | 26.11 | ± | 0.14 |  | 1.23 | ± | 0.07 | 2.92 | ± | 0.06 |
| **3h** | 0.66 | ± | 0.32 | 1.77 | ± | 0.20 |  | 1.02 | ± | 0.05 | 1.15 | ± | 0.15 |
| **6h** | 1.68 | ± | 0.17 | 0.85 | ± | 0.09 |  | 0.73 | ± | 0.11 | 0.72 | ± | 1.03 |
| **24h** | 0.89 | ± | 0.21 | 0.83 | ± | 0.42 |  | 1.88 | ± | 0.15 | 4.79 | ± | 0.43 |
| **30h** | 1.75 | ± | 0.13 | 1.17 | ± | 0.20 |  | 2.48 | ± | 0.11 | 16.11 | ± | 0.36 |
| **48h** | 1.00 | ± | 0.00 | 1.77 | ± | 0.00 |  | 1.32 | ± | 0.00 | 6.70 | ± | 0.00 |
|  |  |  |  |  |  |  |  |  |  |  |  |  |  |
| **-arg** |  |  |  |  |  |  |  |  |  |  |  |  |  |
| **0h** | 1.00 | ± | 0.09 | 1.00 | ± | 0.08 |  | 1.00 | ± | 0.11 | 1.00 | ± | 0.10 |
| **1.5h** | 1.02 | ± | 0.14 | 3.73 | ± | 0.53 |  | 1.43 | ± | 0.16 | 12.64 | ± | 1.31 |
| **3h** | 1.29 | ± | 0.19 | 3.94 | ± | 0.27 |  | 1.30 | ± | 0.09 | 14.26 | ± | 1.13 |
| **6h** | 2.11 | ± | 0.22 | 2.87 | ± | 0.24 |  | 2.20 | ± | 0.14 | 8.06 | ± | 0.20 |
| **24h** | 1.81 | ± | 0.20 | 1.55 | ± | 0.13 |  | 2.32 | ± | 0.21 | 9.53 | ± | 0.68 |
| **30h** | 4.14 | ± | 0.60 | 2.42 | ± | 1.03 |  | 4.72 | ± | 0.55 | 11.90 | ± | 1.11 |
| **48h** | 2.68 | ± | 0.50 | 2.39 | ± | 0.60 |  | 3.67 | ± | 0.38 | 22.26 | ± | 1.55 |
